# Overexpression of the *Trichoderma brevicompactum tri5* Gene: Effect on the Expression of the Trichodermin Biosynthetic Genes and on Tomato Seedlings

**DOI:** 10.3390/toxins3091220

**Published:** 2011-09-23

**Authors:** Anamariela Tijerino, Rosa Hermosa, Rosa E. Cardoza, Javier Moraga, Monica G. Malmierca, Josefina Aleu, Isidro G. Collado, Enrique Monte, Santiago Gutierrez

**Affiliations:** 1 Spanish-Portuguese Center of Agricultural Research (CIALE), Department of Microbiology and Genetics, University of Salamanca, Campus of Villamayor, Río Duero 12, 37185 Salamanca, Spain; Email: atijerino@usal.es (A.T.); rhp@usal.es (R.H.); emv@usal.es (E.M.); 2 University of León, Campus of Ponferrada, Superior and Technical Universitary School of Agricultural Engineers, Area of Microbiology, Avda. Astorga s/n, 24400 Ponferrada, Spain; Email: recars@unileon.es (R.E.C.); mgomh@unileon.es (M.G.M.); 3 University of Cádiz, Puerto Real, Science Faculty, Department of Organic Chemistry, 11510 Cádiz, Spain; Email: javier.moraga@uca.es (J.M.); josefina.aleu@uca.es (J.A.); isidro.gonzalez@uca.es (I.G.C.)

**Keywords:** trichothecene, terpene, tyrosol, * tri* genes, ergosterol, gene expression

## Abstract

*Trichoderma brevicompactum* IBT 40841 produces trichodermin, a trichothecene-type toxin that shares most of the steps of its biosynthesis with harzianum A, another trichothecene produced by several *Trichoderma* species. The first specific step in the trichothecene biosynthesis is carried out by a terpene cylcase, trichodiene synthase, that catalyzes the conversion of farnesyl pyrophosphate to trichodiene and that is encoded by the *tri5* gene. Overexpression of *tri5* resulted in increased levels of trichodermin production, but also in an increase in tyrosol and hydroxytyrosol production, two antioxidant compounds that may play a regulatory role in trichothecene biosynthesis, and also in a higher expression of three trichothecene genes, *tri4*, *tri6* and *tri10*, and of the *erg1* gene, which participates in the synthesis of triterpenes. The effect of *tri5* overexpression on tomato seedling disease response was also studied.

## 1. Introduction

*Trichoderma* species are well known as biocontrol agents of plant diseases in a broad variety of crops [1], and their ability to produce a huge diversity of antimicrobial compounds and cell wall degrading enzymes has been extensively studied and reviewed [2,3,4,5]. Among the secondary metabolites produced by *Trichoderma*, the terpenes include hundreds of different compounds with a huge diversity of physiological activities. Terpenes arise from the repetitive fusion of branched five-carbon units based on an isopentane skeleton, and most of the chemical intermediates in their biosynthetic pathway are known. In fungi, the first enzyme involved in the terpene pathway is 3-hydroxy-3-methylglutaryl-coenzyme A reductase (HMG-CoA reductase), which metabolizes HMG-CoA to mevalonic acid (MVA). Subsequently, the isomerization of mevalonate produces isopentenyl diphosphate (IPP), which condenses with dimethylallyl diphosphate to produce geranyl diphosphate (GPP), the key precursor of monoterpene biosynthesis. This molecule is condensed again with IPP, affording farnesyl diphosphate (FPP), a common intermediate in the production of: (i) geranylgeranyl diphosphate (GGPP) and its diterpene derivatives; (ii) trichodiene and the subsequent sesquiterpene compounds, including trichothecene fungal toxins; and (iii) squalene, as a precursor of the triterpene pathway to produce prenylated proteins, sterols (ergosterol), ubiquinones, dolichols and other secondary metabolites [6,7] as well as plant carotenoids, gibberellins, tocopherols and chlorophylls [8].

The biosynthesis of trichothecenes has mainly been documented in *Fusarium* species, and their biosynthesis has been extensively reviewed [9,10]. Recently, most of the steps involved in the trichothecene biosynthesis in *Trichoderma* species have been described and the genes characterized [11]. *Trichoderma brevicompactum* has been classified within the clade *Brevicompactum* [12], which includes species with a common pathway from FPP to trichodermol. From trichodermol the pathway diverges to produce harzianum A in *T. arundinaceum*, *T. turrialbense* and *T. protrudens* or trichodermin, in *T. brevicompactum*. The *tri5* gene, involved in the biosynthesis of trichodiene from FPP, has recently been cloned and characterized [11,13]. Additionally, another 7 genes involved in trichodermin biosynthesis have been identified in the *T. brevicompactum* genome. These genes are grouped in a *core cluster* [11]. Interestingly, the *tri5* gene is located outside this cluster, being an exception of the trichothecene biosynthetic clusters described until now in *Fusarium* [14] and *Myrothecium* [15] where *tri5* gene is located inside the clusters.

Transformants of *T. brevicompactum* IBT 40841, with a higher level of *tri5* gene expression, had higher levels of trichodermin production and greater antibiotic activity against several yeast strains. A significant increase in the level of production of the antioxidants tyrosol and hydroxytyrosol was also detected in the *tri5*-overexpressing transformants [13]. Tyrosol is a phenolic antioxidant metabolite that is present in olive oil and wine [16], yeast [17], and *T. viride* [18] and *T. brevicompactum* cultures [13]. It is synthesized from a transamination reaction of L-tyrosine and 2-oxoglutarate, giving rise to L-glutamic acid and *p*-hydroxyphenyl-pyruvate. The later product is then decarboxylated, and the resulting aldehyde is reduced to tyrosol [19,20,21]. It has been shown that the tyrosol acts as a quorum-sensing molecule, which accelerates the morphological transition from yeasts to hyphae and facilitates biofilm production [17]. Hydroxytyrosol is a derivative of tyrosol with antioxidant properties due to its ortho-dihydroxyphenolic group [19]. The relationship between the tyrosol/hydroxytyrosol and trichodermin is not clear and needs additional studies. 

In the present article, we report the effect of *tri5* gene overexpression on the expression of other *tri* genes in minimal medium and in media containing the antioxidant tyrosol. The effect of *tri5* overexpression on expression of the *erg1* gene, which is involved in the synthesis of triterpene compounds [22], was also determined. Finally, the effect of *tri5* overexpression on tomato seedling disease response was examined.

## 2. Materials and Methods

### 2.1. Fungal Strains

*T. brevicompactum* IBT 40841 (=IBT40841) (IBT Culture Collection, Department of Biotechnology, Technical University of Denmarck, Kongens Lyngby, Denmark) wild type strain and three *tri5-*overexpressing transformants Tb38tri5, Tb40tri5 and Tb41tri5 [13] were used throughout this study. These strains were maintained on potato dextrose agar medium (PDA) (Difco Becton Dickinson, Sparks, MD, USA).

### 2.2. Media and Culture Conditions

*T. brevicompactum* mycelia were obtained following a two-step liquid culture procedure [23]. First, the fungus was grown in potato dextrose broth medium (PDB) (Difco Becton Dickinson, Sparks, MD, USA) at 28 °C for 36 h and 150 rpm. The fungal biomass was harvested by filtration through 30 μm pore diameter nylon filters (Sefar AG, Heiden, Switzerland), and the mycelium transferred to minimal medium (MM) [24], containing 20 g/L glucose and/or supplemented with 0.25 or 5 mM of the antioxidant tyrosol when indicated. Cultures were maintained at 28 °C on a rotary shaker at 150 rpm for 1 or 3 days. Biomass was recovered by filtration, washed twice with sterile saline solution (9 g/L NaCl), frozen in liquid nitrogen, and stored at −80 °C until RNA extraction.

### 2.3. DNA and RNA Manipulations

Standard molecular techniques were performed throughout [25]. RNA extraction was carried out using the TRIZOL reagent (Invitrogen Life Technologies, Carlsbad, CA, USA) according to the manufacturer’s instructions from 30 mg mycelium. The integrity and pureness of the RNA was determined using the Agilent 2100 Bioanalyzer (Agilent Technologies, Santa Clara, CA, USA) and cDNA was synthesized with Superscript Choice System for cDNA (Invitrogen Life Technologies, Carslbad, CA, USA) using the Reverse Transcription System (Promega, Madison, WI, USA) and then quantified by determining the absorbance at 260 nm on a spectrophotometer.

### 2.4. Gene Expression Studies

Quantitative real-time PCR was performed using the Step One Plus^TM^ system (Applied Biosystems, Foster City, CA, USA) with Brilliant SYBR Green QPCR Master Mix (Stratagene, La Jolla, CA, USA). All PCR reactions were performed in triplicate in a total volume of 12 μL for 40 cycles under the following conditions: denaturation, 95 °C, 15 s; annealing, 60 °C, 1 min; extension, 72 °C, 1 min; and final extension, 72 °C, 3 min. Two different sets of cDNA were used for each sample. Threshold cycles (CT) were determined using the 7000 SDS System Software, and CT values were calculated using the β*-tubulin* gene as housekeeping control. Data are expressed as 2^−^^ΔΔ^^CT^ [26]. Six values per sample were used for statistical analysis. The primers used in this work are shown in Table 1.

**Table 1:** Oligonucleotides used in the Real-Time PCR experiments.

Gene	Name	Sequence (5´-3´)
*erg1*	erg1-f	CGCTCCGTGCTTCTTCTC
*erg1*	erg1-r	CTTCTTCTCTCCCGTCTCC
*tri3*	Tri3-F	CATTCAGCCACCTAACCTAACCG
*tri3*	Tri3-R	CCATCCTTCAACCACCGTCGGC
*tri4*	Tri4-F	CTTGATGGAGCCTTCTCAGC
*tri4*	Tri4-R	CATCAAGATAGTCCTTATGTTC
*tri6*	Tri6-F	CGTGCTGACGTGGTTCGAGTGC
*tri6*	Tri6-R	CTATGGAATGGGTCGGCGAATC
*tri10*	Tri10-F	CGCTCTCATATGAGTACGTTGGC
*tri10*	Tri10-R	CCATGAATGGTGAAGATGGGC
*tri11*	Tri11-F	CGCGAGTACGCTTATTACCG
*tri11*	Tri11-R	GCAGAGCGCACTTCTTCAGTC
*tri12*	Tri12-F	GTTCCATATCTTCCGCCATATTC
*tri12*	Tri12-R	GCGATTGACAGAAGCCATTGC
*tri14*	Tri14-F	GCTGATGCTGAGCTTGCAAGTG
*tri14*	Tri14-R	GCCAAGAGGCTCTTGGACGAAG
*β-Tubulin*	T-tub-F	GAATATCAACAATACCAGGATGG
*β-Tubulin*	T-tub-R	AGGATTGGTATTGATCATCAGCA

### 2.5. Tomato Seedling Assay

The effect of *Trichoderma* strains (wild-type and *tri5-*overexpressing transformants) on tomato seedlings was evaluated *in vitro*. Fungal spore densities of 1 × 10^6^ spores were inoculated by placing the spores at the opposite end of Murashige and Skoog (MS) medium plates (Duchefa Biochemie B.V., Haarlem, The Netherlands), supplemented with 1% (w/v) sucrose and 8 g/L agar, pH 5.7, containing 3-day-old germinated tomato seedlings (5 seedlings per plate). Plates were sealed with flexible film (Parafilm) (Pechiney, Menasha, WI, USA), placed vertically to allow root growth along the agar surface and to allow unimpeded aerial growth of the hypocotyls, and cultured in a growth chamber under conditions of 40% humidity, 24 °C, and a 16 h light/8 h dark photoperiod at 80–100 µE m^−2^ s^−1^. MS plates, containing only tomato seedlings, without *Trichoderma* spores were used as control. Experiments were performed in triplicate and measurements of plant size, main root length and number of lateral roots were taken 10-days after *Trichoderma* inoculation.

## 3. Results

### 3.1. Effect of *tri5* Gene Overexpression on the Expression of Trichodermin Biosynthetic Genes

The expression of seven genes (*tri4*, *tri3*, *tri11*, *tri6*, *tri10*, *tri12* and *tri14*) of the trichodermin biosynthetic pathway was analyzed from RNAs extracted from mycelia of the wild-type strain IBT40841 and three selected transformants overexpressing the *tri5* gene: Tb38tri5, Tb40tri5 and Tb41tri5, incubated 3 days in MM [24] supplemented with 20 g/L glucose as described above.

**Figure 1:**
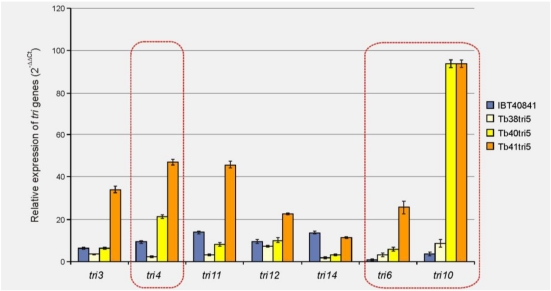
RT-PCR quantification of expression of seven genes from the trichothecene biosynthetic pathway (*tri* genes) in the wild-type strain IBT40841 and three *tri5*-overexpressing transformants Tb38tri5, tb40tri5 and Tb41tri5. The basal condition used for relative measurements was MM without glucose (2^−ΔΔCt^ = 1). IBT40841 β*-tubulin* was used as an internal reference gene.

The highest levels of expression were observed in the Tb41tri5 transformant, a strain that produces 2.8-folds more trichodermin than the wild type strain [13]. Expression of *tri14* was slightly lower in this transformant that in the wild-type strain (Figure 1).

The expression values of the *tri3*, *tri4*, *tri11* and *tri12* genes in the Tb38tri5 transformant were similar to or lower than those of the wild-type strain. No large differences were observed in the expression levels of the *tri3* and *tri12* genes between the wild-type and the Tb40tri5 strains. However, the *tri11* and *tri14* genes showed a lower level of expression, and the *tri4* gene showed a considerably higher level of expression in Tb40tri5 than in the wild-type strain. In the case of the regulatory genes, *tri6* and *tri10*, the level of expression was higher in all the transformants than in the wild-type strain, the greatest differences being seen in the values of *tri10* gene expression between the wild-type and the Tb40tri5 and Tb41tri5 transformants. It was previously observed under identical growth conditions that the transformants Tb38tri5, Tb40tri5 and Tb41tri5 showed higher *tri5* transcript levels than the wild-type strain [13], and although the transformation cassette had been inserted twice into the genomes of the three *tri5*-overexpressing transformants, they were inserted in different genome locations in the different transformants.

### 3.2. Effect of Tyrosol on the Expression of the *tri4*, *tri6* and *tri10* Genes in the *tri5* Overexpressing Transformants

**Figure 2:**
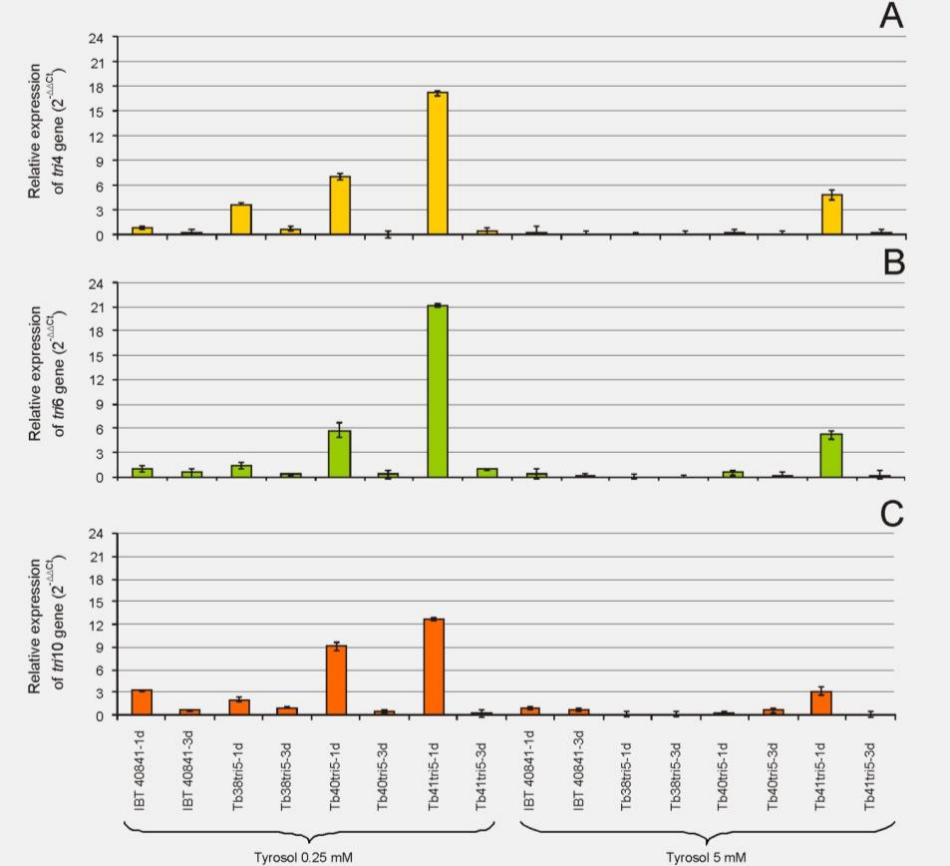
RT-PCR quantification of (**A**) *tri4*, (**B**) *tri6* and (**C**) *tri10* gene expression in the wild-type strain and three *tri5* overexpressing transformants. The experiment was carried out with mycelia grown for 1 or 3 days in MM [24], with 0.25 or 5 mM tyrosol. The basal condition and the internal reference genes used were those described in Figure 1.

The different strains were grown for 1 or 3 days in MM [24], with 20 g/L glucose, supplemented with 0.25 or 5 mM of the antioxidant tyrosol. The pattern of expression of the *tri4*, *tri6* and *tri10* genes was similar for the two tyrosol concentrations (Figure 2).

In general, all the genes were repressed by the addition of tyrosol in comparison with the use of MM lacking tyrosol. All the genes showed a higher level of expression after 1 day of growth. In addition, the expression of the genes was higher when the lower concentration of tyrosol was used. The greatest differences in the expression levels were observed between the wild-type strain and the transformants Tb40tri5 and Tb41tri5 after 1 day of growth in the presence of 0.25 mM tyrosol.

### 3.3. Effect of the Overexpression of the *Tbtri5* Gene in the Expression of *erg1* Gene

**Figure 3:**
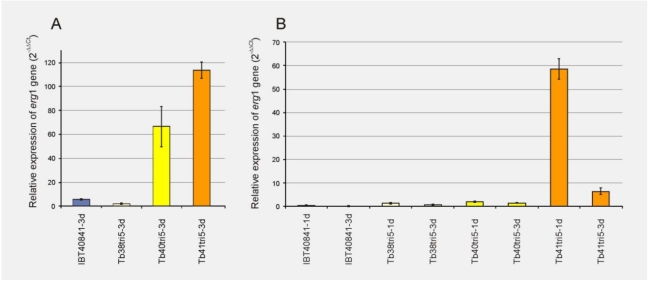
RT-PCR quantification of the *erg1* transcript, in the wild-type strain IBT40841 and three *tri5* overexpressing transformants. The strains were grown (**A**) for 3 days in MM [24], supplemented with 20 g/L glucose or (**B**) for 1 or 3 days in MM, supplemented with 20 g/L glucose and 0.25 mM tyrosol. The basal condition and the internal reference genes used were those described in Figure 1.

The *erg1* gene encodes the enzyme squalene epoxidase, which is involved in the biosynthesis of triterpenes [22]. These compounds are synthesized through a pathway common to the trichothecenes up to FPP, from which the pathway diverges to give rise to diterpenes, sesquiterpenes (*i.e.*, trichothecenes) and triterpenes (*i.e.*, ergosterol). The expression levels of *erg1* were analyzed by real-time PCR from mycelia grown over 3 days in MM supplemented with 20 g/L glucose. Higher levels of expression of the *erg1* gene were found in the Tb40tri5 and Tb41tri5 transformants in comparison with the wild-type strain (Figure 3A).

Finally, the effect of the antioxidant agent tyrosol on the expression of *erg1* gene was analyzed in the wild-type and in the *tri5* overexpressing strains. Thus, the different strains were grown for 1 or 3 days in MM, supplemented with 20 g/L glucose and 0.25 mM tyrosol. The expression levels showed that in all the strains assayed, expression of the *erg1* gene was reduced in the presence of tyrosol. The greatest differences in *erg1* expression levels were found in the Tb40tri5 and Tb41tri5 transformants grown in the presence of 0.25 mM tyrosol in comparison with those transformants grown without tyrosol its absence (Figure 3B).

### 3.4. Effect of the *tri5* Overexpression on Tomato Seedlings

An *in vitro* assay was performed to determine the effect of *tri5* overexpression on the development of tomato seedlings, using the wild-type strain and the three *tri5*-overexpressing transformants.

Ten days after the inoculation of plates containing 3-day-old germinated tomato seedlings with the different *Trichoderma* strains or with water (control), three parameters were measured for each condition: plant size, length of the main root and number of lateral roots.

The mean value of plant sizes was 4.1 ± 1 cm when they were grown in the presence of water (control); 4 ± 0.6 cm in the presence of the wild type strain; 3.7 ± 0.5 cm with Tb38tri5; 3.8 ± 1 cm with Tb40tri5 and 3.6 ± 0.4 cm with Tb41tri5. Statistically significant differences were observed between the size of the control plants and those growing in the presence of the three transformants (*p* = 0.05), and also, between the size of the plants growing in the presence of the wild-type strain and those grown in the presence of Tb38tri5 (*p* = 0.046) and Tb41tri5 (*p* = 0.045). The data indicated that both the presence of *T. brevicompactum* in the growth medium and the *tri5* gene overexpression negatively affected to the size of the tomato seedlings.

**Figure 4:**
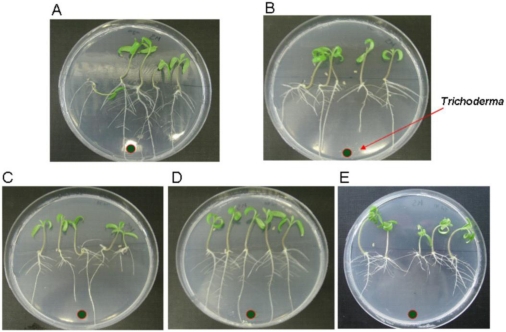
Effect of *T. brevicompactum* and of *tri5* overexpressed transformants on tomato seedlings. Three-day-old germinated tomato seedlings grown on MS were inoculated with (**A**) water; (**B**) IBT40841; (**C**) Tb38tri5; (**D**) Tb40tri5; or (**E**) Tb41tri5. Photographs were taken 10-days after inoculating 1 × 10^6^ *Trichoderma* spores.

Root length was also lower when the tomato seedlings were grown in the presence of IBT40841 or the *tri5*-overexpressing transformants in comparison with the control condition without *Trichoderma* in the medium. The mean values were: 6.6 ± 2.0 cm in the control; 5.1 ± 1.0 cm with the *Tb41* strain; 4.8 ± 0.8 cm with Tb38tri5; 5.9 ± 1.2 cm with Tb40tri5; and 5.6 ± 1.0 cm with Tb41tri5. Finally, it was observed that the number of lateral roots, in comparison with the control, was similar or was reduced when wild-type IBT40841 or the *tri5*-overexpressing transformants were present in the medium used to grow the tomato seedlings. The mean values were: 15.9 ± 8.0 in the control, 11.5 ± 5.2 with the wild type strain, 11.1 ± 4.2 with Tb38tri5, 15.9 ± 4.6 with Tb40tri5 and 14.8 ± 3.0 with Tb41tri5. The greatest reduction in the number of lateral roots with respect to the control was seen in the presence of the wild-type strain or the Tb38tri5 transformant (Figure 4). In both determinations (root length and number of lateral roots), statistically significant differences were seen between the control condition and the conditions that contained *Trichoderma* (*p*≤ 0.05), and also between the condition treated with the wild-type strain and those treated with spores from the three *tri5*-overexpressing transformants (*p* = 0.05) (Figure 4).

## 4. Discussion

Overexpression of the IBT40841 *tri5* gene resulted in an increase of the trichodermin production as a result of a higher level of *tri5* transcript and also in an increase in the antibiotic activity against a large panel of yeast, and it negatively affected tomato plant growth and the lesions caused by *Botrytis cinerea* [13].

**Figure 5:**
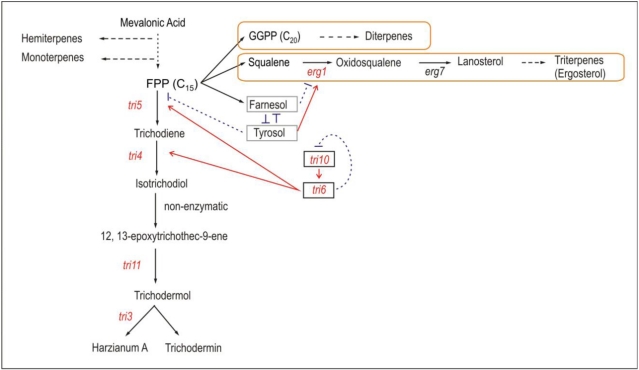
Schematic representation of the model of regulation of the trichothecenes harzianum A and trichodermin biosynthesis. The synthesis of diterpenes and triterpenes is indicated by yellow squares. The genes indicated in red are those studied in the present work. *tri6* and *tri10* are regulatoy genes. Farnesol and tyrosol are two regulatory and antagonistic molecules. The red arrows indicated positive regulation. The blue doted lines indicate possible inhibitory activities (Modified from [12,27,28,29,30]).

Two of the *tri5* gene overexpression transformants used in the present work, Tb40tri5 and Tb41tri5, had an increased level of expression of the regulatory genes *tri6* and *tri10*, and *tri4*, the oxygenase gene that controls conversion of trichodiene to trichodermol [11]. However, in the Tb41tri5 transformant, the expression of *tri3*, *tri11* and *tri12* genes were also induced. In spite of these differences between the different transformants it is likely that the overproduction of trichodiene has a positive effect on the expression of the *tri4* gene, the next gene in the biosynthetic pathway (Figure 5), but also in the expression of the *tri6* and *tri10* genes, that would regulate the expression of all the *tri* genes.

The production of tyrosol and hydroxytyrosol simultaneous to the increase of the trichodermin level in the *tri5*-overexpressing transformants can be explained as a response by cells to regulate the levels of farnesol:tyrosol, since both trichodiene and farnesol have FPP as precursor. When farnesol and tyrosol are present in direct competition, tyrosol does not alter the quorum-sensing activity of farnesol, even when the tyrosol is present at a 16-fold molar excess [31]. When tyrosol was added to the culture media, the expression of the *tri5*, *tri4*, *tri6* and *tri10* genes was repressed, and this repressing effect persisted until at least the seventh day of culture (data not shown). However, this repressing effect of tyrosol on *tri* gene expression was observed only when this compound was added to the culture medium but when tyrosol was produced by the *tri5*-overexpressing transformants, rather, there was a 2.4 fold increase in toxin production in the transformant Tb41tri5 when compared to the wild-type strain [13]. Thus, without external addition of tyrosol a significant increase in the expression of the *tri* genes was observed in the transformants (see Figure 1), probably because the effect of the endogenous tyrosol would be regulated by the intracellular farnesol, equilibrating both stimulating and repressing activities, respectively. The addition of external tyrosol would shift this equilibrium towards the stimulating effects of the tyrosol, resulting in a repression of the genes involved in the biosynthesis of trichodermin, as a secondary metabolite, and a stimulation of the primary metabolism biosynthetic pathways. In this sense, the increase in the level of expression of the *erg1* gene in the *tri5* overexpressing transformants can be understood in the same way, since *erg1* encodes squalene epoxidase, the enzyme that uses squalene as substrate in the first step of the ergosterol biosynthesis. Squalene is the condensation product of two farnesyl pyrophophates. Thus, the increase in the level of expression of *erg1* gene will direct the FPP towards the ergosterol biosynthesis, which is needed for the fungal cell growth, one of the stimulating effects exerted by the tyrosol. This correlates well with the previous observation that farnesol biosynthesis inhibits the ergosterol pathway in *C. albicans* [32].

Many *Trichoderma* species maintain a relationship with the plants as avirulent symbionts [33], with benefits that include better root development, increase in crop production and defense stimulation [34]. Secondary metabolites produced by *Trichoderma* have stimulant effects on plant development when they are applied at low doses [35]. However, it has been described that *Fusarium* spp. trichothecenes are phytotoxic and that they act as virulence factors in host-compatible plants [36]. Furthermore, it is known that trichothecenes such as harzianum A and trichodermin, characteristic from the clade * Brevicompactum*, are strong inhibitors of the seed germination and that they do not promote plant growth owing to their phytotoxicity [12,37,38]. The *in vitro* assays carried out in the present work with tomato seedlings showed that the plant size and the root length were lower in the presence of *T. brevicompactum* and even lower with the Tb40tri5 and Tb41tri5 transformants. In addition, it has recently been shown in greenhouse assays that tomato seed treatments with *T. brevicompactum* strains increased the leaf necrotic lesions produced by *B. cinerea* after leaf inoculations with this pathogen, and mainly when the tomato seeds were coated with the *tri5*-overexpressing transformants, invalidating *T. brevicompactum* IBT 40841 as a biocontrol agent in agriculture [13].

## 5. Conclusions

In this work we have shown that the overexpression of the *tri5* gene resulted in an increase of the level of transcription of three trichothecene genes, *tri4*, *tri6* and *tri10* genes, and that *tri5* gene may have an important role in the trichothecene-triterpene-diterpene regulatory network, since *tri5* expression may contribute to the balance of FPP-farnesol-squalene-trichodiene in the different cell growth stages. We found that growth in the presence of added tyrosol reduced the *tri* gene expression and increased *erg1* gene expression, directing the pool of FPP towards biosynthesis of ergosterol, a primary metabolite, instead of towards the synthesis of secondary metabolites like trichothecenes, thus avoiding the repressing effect exerted by farnesol. Finally, transformants Tb40tri5 and Tb41tri5 reduced plant growth and main root size to a greater extent than the wild type strain, probably due to the intrinsic phytotoxic effect of the trichodermin, whose production was significantly increased in these transformants.
